# Directed evolution of a transcription factor PbrR to improve lead selectivity and reduce zinc interference through dual selection

**DOI:** 10.1186/s13568-020-01004-8

**Published:** 2020-04-10

**Authors:** Xiaoqiang Jia, Yubing Ma, Rongrong Bu, Tingting Zhao, Kang Wu

**Affiliations:** 1 Department of Biochemical Engineering, School of Chemical Engineering and Technology, Tianjin University, Tianjin, 300072 People’s Republic of China; 2 Frontier Science Center for Synthetic Biology and Key Laboratory of Systems Bioengineering (MOE), School of Chemical Engineering and Technology, Tianjin University, Tianjin, 300350 People’s Republic of China; 3 Collaborative Innovation Center of Chemical Science and Engineering (Tianjin), Tianjin, 300072 People’s Republic of China; 4 Department of Chemical Engineering, University of New Hampshire, Durham, NH 03824 USA

**Keywords:** Transcription factor, PbrR, Dual selection system, Directed evolution, Specificity

## Abstract

Directed evolution has been proven as a powerful tool for developing proteins and strains with novel or enhanced features. In this study, a dual selection system was designed to tune the binding specificity of a transcription factor to a particular ligand with the ampicillin resistance gene *amp* (ON selection) as the positive selection marker and the levansucrase gene *sacB* (OFF selection) as the negative selection marker. It was applied to the lead responsive transcription factor PbrR in a whole-cell lead biosensor previously constructed in our lab (Jia et al. in Fems Microbiol Lett 365:fny157, 2018). After multiple rounds of ON–OFF selection, two mutants with higher specificity for lead were selected. Structural analysis revealed that the mutation C134 located on the metal-binding loop at the C-terminal of PbrR is likely associated with the enhanced binding to both lead and cadmium. The double mutations D64A and L68S close to the metal-binding residue C79 may lead to the reduced binding specificity toward zinc ions. This dual selection system can be applied to engineer the specificity of other transcription factors and provide fine-tuned tools to synthetic biology.

## Introduction

In recent years, directed evolution has been widely used for the development and the improvement of enzymes and proteins (Currin et al. 2015). The key to directed evolution lies in the construction of the mutant library and the efficiency of the screening process. At present, methods for constructing mutant libraries are well established. Developing efficient screening strategies has become the bottleneck of protein directed evolution technology (Cadwell and Joyce 1992; Stemmer 1994; Zhao et al. 1998). Traditional fluorescence activated cell sorting (FACS) screening methods have been applied to the directed evolution of transcriptional regulatory proteins, but this method requires expensive instrumentation and does not validate evolutionary results in real time (Hakkila et al. 2011). Yokobayashi et al. designed a dual genetic screening module to select a genetic inverter from a 200-fold excess of nonfunctional inverters in two rounds (Yokobayashi and Arnold 2005). The key to this design was to couple cell survival with the desired feature and maintain the evolution of the gene circuits in host cells. In this study, we designed a dual selection module to tune the specificity of a transcription factor toward a particular inducer and select the mutants that are only respond to the target inducer so that it can minimize the response to other competing inducers. In presence of the target inducer (ON selection), the mutant libraries are evolved and selected with the positive marker, the ampicillin resistance gene *amp*, and only the ones that can be activated by the target inducer can survive. In presence of the competing inducers (OFF selection), the library will be selected using the negative marker, the levansucrase gene *sacB*, and only the ones that do not respond to these inducers can survive. By alternating these ON–OFF selection steps for multiple cycles, mutant regulators only in response to the target inducer are expected to be obtained. This strategy can be applied to enhance the specificity of transcription factor based biosensors in a way that it strengthens the signal of the biosensors in response to the target analyte and minimizes the noise.

Lead is a bioaccumulative and highly toxic heavy metal that can cause serious damage to the ecological environment and human health (Bai et al. 2015). Under normal circumstances, the lead concentration in the human body should be less than 0.1 mg L^−1^, and once the lead concentration exceeds the standard, it will quickly affect the nervous system and growth and development, leading to the occurrence of lead poisoning (Shen et al. 1998). Lead pollution in the environment is the main cause of frequent lead poisoning incidents (Baker et al. 1977; Oliver 1911). Therefore, the development of rapid and efficient methods to detect the lead ions concentration in the environment has become the key to the prevention and control of lead pollution. The determination of the lead concentration in the environment requires advanced chemical equipment and technical expertise, which will result in the inability to detect lead ions in real time in some areas (Badiei et al. 2013; Oliveira et al. 2011; Zhao et al. 2009). To solve this problem, biosensors which are simpler and less expensive than analytical instruments, and are valuable for in situ detection of lead are being used (Liao et al. 2006; Qu et al. 2016).

The use of whole-cell biosensors to determine heavy metal concentrations has been reported, and the biosensors use microbial live cells as biometric materials identify and detect substances to be tested (Aleksic et al. 2007; Tauriainen et al. 1998). At present, almost all whole-cell lead biosensors use the transcription factor PbrR from the plasmid pMOL30 of the bacterium *Ralstonia metallidurans* CH34 as the sensing element (Mergeay et al. 2003; Monchy et al. 2006). The lead responsive transcription factor PbrR belonging to the MerR family activates transcription upon binding to lead ions (Hobman et al. 2012). Due to the structural similarity of the MerR family transcription factors, many of them can respond to multiple divalent ions. PbrR is the most specific regulator in response to the lead ions. Still, it is known to respond to other ions like zinc, copper, mercury, etc. (Angeli et al. 2004). When testing real samples, often the output signal is attributed to divalent ions as a whole, and not just lead. It is speculated that the unique physicochemical properties of the lead ions and the protein conformation of metal binding domain may be the main factors affecting the specific binding of lead ions to PbrR. As reported, the cysteine residues in regulator play a significant role in coordinating with metal ions and activating the expression of the gene that is downstream of the promoter (Monchy et al. 2006; Shewchuk et al. 1989). With limited information on the structure of PbrR, rational design of a mutant of desired function is impossible and directed evolution offers a potential solution in this situation (Bornscheuer et al. 2012; Cobb et al. 2013). However, traditional screening strategy in directed evolution does not work well to improve the specificity of these regulators. Therefore, the dual selection system was applied to improve the screening efficiency by exerting both positive and negative selection pressures.

The aim of our study was to enhance the specificity of PbrR toward lead and to mitigate the interference of the divalent metal ions zinc. To achieve this goal, we created a mutant library by error-prone PCR and evolved and selected the desired PbrR mutants with the dual selection system. Compared with the wild type, the mutant strains M1 and M2 had increased response to the lead ion with 1.8-fold and 2-fold respectively. In addition, the wild-type growth was inhibited during the OFF selection with zinc ions, while the mutant strain M1/M2 rapidly grew, and weakened zinc-binding ability was observed. Structural simulations indicated that the mutation C134R of M1 was located on the C-terminal metal-binding loop region, which may lead to the enhancement of cadmium ion binding, and the double mutations D64A and L68S of M2 were located on the α-helix α4 near the loop region of C79. Amino acid mutations near the metal binding domain of the dimeric protein may cause subtle force changes and spatial changes, leading to reduced binding capacity of zinc ions.

## Materials and methods

### Bacterial strains, reagents, and growth conditions

Construction and characterization of the designed selection plasmid were performed in *Escherichia coli* DH5α. Cells were grown in Luria–Bertani (LB) broth (10 g L^−1^ peptone, 5 g L^−1^ NaCl, 5 g L^−1^ yeast extract) containing 50 μg mL^−1^ kanamycin. Solid plates were made using the same medium with 1.5% (w/v) agar. The lead ion was added at a final concentration of 50 µM and ampicillin was added at a final concentration of 100 μg mL^−1^ during the ON selection stage. Metal ions were added at a final concentration of 50 µM during the OFF selection stage. All of the experiments were performed at 37 °C unless otherwise noted.

PCR reagents, restriction endonucleases, and T4 DNA ligases were purchased from TransGen Biotech. Pb(NO_3_)_2_, ZnCl_2_, CuCl_2_, and CdCl_2_ were purchased from Shandong Western Chemical Industry Co. Ltd., China. Oligo primer synthesis and sequencing were performed by GENEWIZ (China).

### Construction of the selection plasmid

The backbone of the selection plasmid (ColE1 Ori-kan) was amplified using the plasmid pZE21 (Lutz and Bujard 1997) as the template and the primers H1F/H1R containing part of the *pbr* operator sequence and part of the OFF selection marker gene sequence. The lead-sensing element *pbrR*-P_*pbrA*_ from *Ralstonia metallidurans* CH34 plasmid pMOL30 (Accession no. NC_006466) (Borremans et al. 2001) was amplified from the plasmid pUC57-G7-kan (Jia et al. 2018) using primers H2F overlapping with the plasmid backbone at the 5′ end and H2R containing part of the positive marker the ampicillin resistance gene *amp*, which was amplified from the plasmid pZE12 (Lutz and Bujard 1997) using the primers H3F/H3R. The OFF selection marker *sacB* was located downstream of the *amp* gene and amplified by PCR using the plasmid pK18mobsacB (Schafer et al. 1994) as the template with the primers H4F/H4R. The PCR products were sequentially joined together to yield the selection plasmid pZE21-PBS. It was confirmed using gel electrophoresis and Sanger sequencing. Strains and plasmids used in this study are listed in Additional file 1: Table S1. Primers are listed in Additional file 1: Table S2.

### Optimization of selection conditions

Cultures of the selected cells (*E. coli* strain DH5α bearing plasmids pZE21-PBS) were grown overnight with agitation in 50 mL fresh LB medium at 37 °C with 50 μg mL^−1^ kanamycin. The overnight culture was used to inoculate 50 mL LB and it was incubated in a shaker until the optical density at 600 nm (OD_600_) reached 0.6.

For selection using ampicillin and lead ions at the ON stage, the overnight culture was diluted 100-fold in 50 mL of fresh LB medium with lead ions at a final concentration of 0, 1, 5, 10, 20, or 50 µM and ampicillin at a final concentration of 100 or 200 μg mL^−1^ and incubated at 37 °C for 24 h with shaking.

For selection using sucrose and zinc ions at the OFF stage, the overnight culture was used as the seed culture to inoculate the subculture by adding 0.5 mL of the seed culture to 50 mL fresh liquid medium containing lead ions of 0, 5, 20, or 50 µM and sucrose at 0, 5, or 10 g L^−1^. The subcultures were incubated at 30 °C for 24 h with shaking.

To verify the expression of the selection genes, the overnight culture was diluted 100-fold in 50 mL fresh liquid medium for the ON selection (LB medium with 100 μg mL^−1^ ampicillin) and 50 mL fresh liquid medium for the OFF selection (LB medium with 10 g L^−1^ sucrose) containing the same concentration of lead and zinc (final concentrations: 20 or 50 µM). The subcultures in the ON selection were incubated at 37 °C while the subcultures in the OFF selection were incubated at 30 °C for 24 h at 220 rpm.

The optical density at 600 nm of the three expression verification systems was measured by a spectrometer (Unico UV-2000, USA) every 3 h after inoculation. All of the samples were measured in triplicates.

### Directed evolution of PbrR

#### Creating the mutant library

The *pbrR* gene was amplified by error-prone PCR from the selection plasmid pZE21-PBS using primers EP2F and EP2R (Additional file 1: Table S2) having the ends overlapping with the plasmid backbone. The primers EPF and EPR (Additional file 1: Table S2) were used to amplify the backbone of the plasmid pZE21-PBS. The two fragments were ligated by homologous recombination and were selected for kanamycin resistance on solid medium.

All of the resistant transformants growing on plates with kanamycin were picked and transferred to the ON selection solid medium, which was incubated at 37 °C, and OFF selection solid medium, which was incubated at 30 °C. There was one-to-one correspondence of transformants on the ON and OFF selection plates. The transformants grown on both ON and OFF selection plates were sequenced using sequencing primer SP12F and SP23R (Additional file 1: Table S2).

#### ON/OFF selection of the mutant strains

Single colonies on the plates were picked and grown in LB medium with kanamycin overnight. Then they were subcultured until the OD_600_ was approximately 0.6. Next, cells were transferred into ON selection liquid medium with lead ions, zinc ions, cadmium ions or copper ions at a final concentration of 50 µM and OFF selection liquid with lead ions, zinc ions at a final concentration of 50 µM. The strain with the wild-type PbrR was used as a control and the OD_600_ of the wild-type and the mutant strains at 5 h and 10 h were recorded.

#### Structure simulation

Analysis of the selected mutants was carried out using the mutagenesis module of PyMOL software, and a partial model of the mutation site of the lead responsive transcription factor PbrR was drawn. The results were analyzed based on the position and structural changes of the model at which the mutation site was located.

## Results

### Construction of the selection plasmid pZE21-PBS

**Fig. 1:**
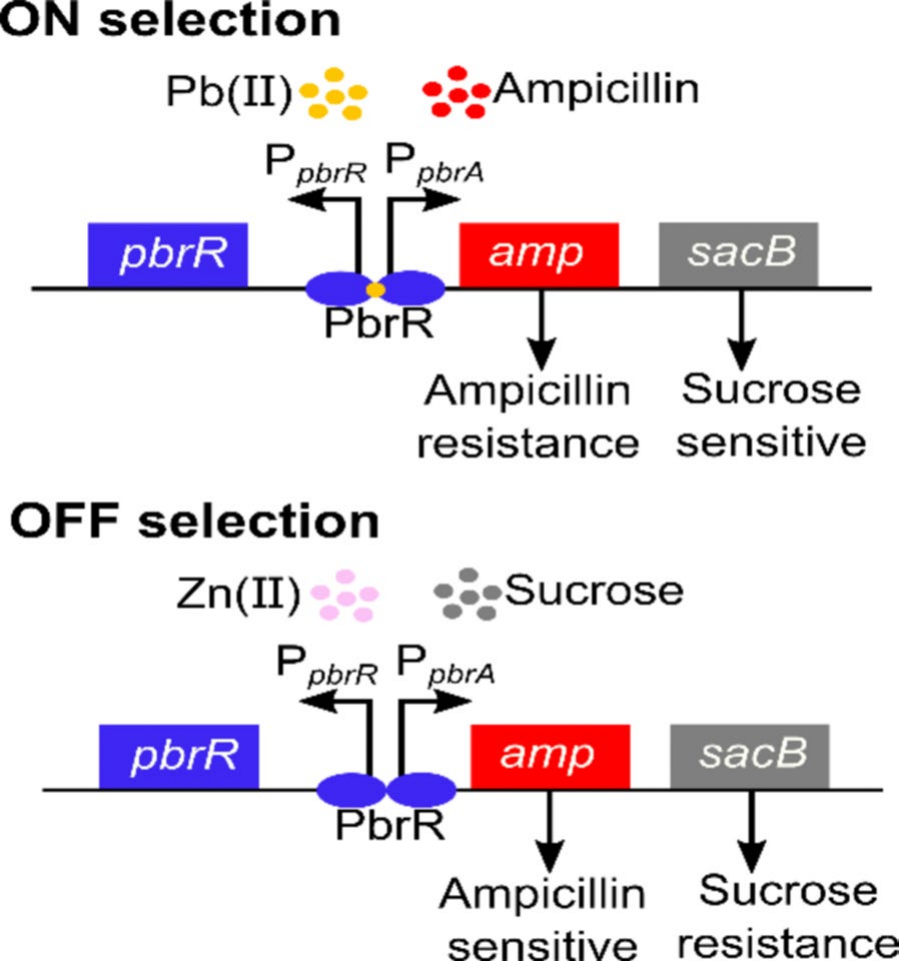
Schematic of the dual selection system for directed evolution of PbrR

As shown in Fig. 1, the positive selection marker gene *amp* and the reverse selection marker gene *sacB* were located downstream of the promoter P_*pbrA*_, and their expression was regulated by PbrR. During the ON selection, cells with PbrR mutants that have strong response to the lead ions and activate the expression of *amp* could survive in presence of ampicillin, while weak binding mutants led to cell death (Fig. 1). At the OFF stage, cells with PbrR mutants that have weaker binding to zinc could survive, because the ones that respond to zinc could switch on the expression of *sacB* which caused cell death in presence of sucrose. The selection plasmid pZE21-PBS was used to confirm the expression of marker genes in the presence of metals and optimize selection conditions.

### Optimization of selection conditions

#### Verification of the expression of amp

The expression of the positive selection marker *amp* was evaluated at different concentrations of lead ions (0, 1, 5, 10, 20, and 50 µM) at 37 °C with ampicillin at a concentration of 100 μg mL^−1^ or 200 μg mL^−1^. The optical density at 600 nm (OD_600_) was measured every 3 h to monitor cell growth.

**Fig. 2:**
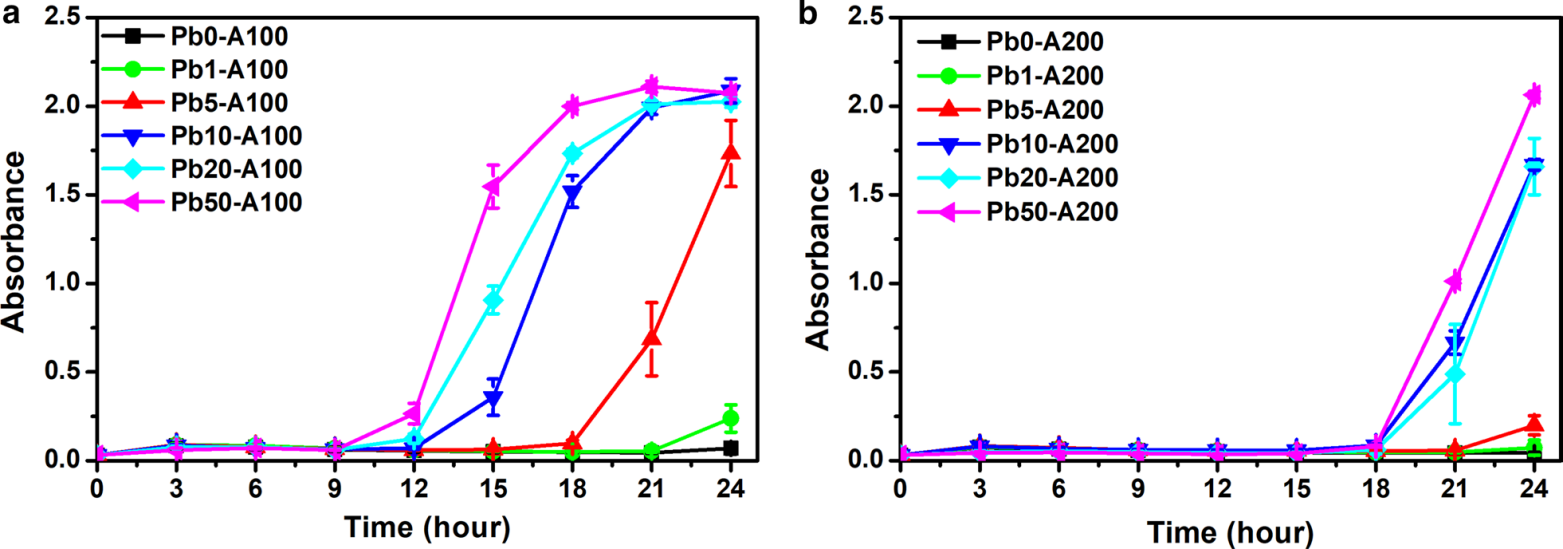
Growth curves of the chassis cells with the selection plasmid in response to lead and ampicillin. The optical density at 600 nm of the *E. coli* (pZE21-PBS) strain was measured with lead at different concentrations of 0 µM (squares), 1 µM (circles), 5 µM (up triangles), 10 µM (down triangles), 20 µM (diamonds), and 50 µM (left triangles). Ampicillin was used at **a** 100 µg mL^−1^ and **b** 200 µg mL^−1^. Experiments were performed in triplicate, and data represent the averages and standard deviations of the means

As shown in Fig. 2a, cells with the selection plasmid did not grow with 100 μg mL^−1^ ampicillin, when the concentration of lead ions was 0 µM. When the concentration of lead was increased to 20 and 50 μM, the cells showed growth after 12 h. A higher level of lead ions elicited a short lag phase and a faster growth rate. Similarly, as shown in Fig. 2b, when ampicillin was used at 200 μg mL^−1^, the cells did not grow without the addition of lead ions. Growth were observed after 21 h when the lead concentration was 10, 20, or 50 μM. The growth of the cells was delayed compared to the growth curve with ampicillin at 100 μg mL^−1^ (Fig. 2a), but the inducer dependent growth was confirmed under both conditions.

#### Verification of the expression of sacB

The expression of the *sacB* gene was evaluated with the cells containing the selection plasmid at different concentrations of the lead ions (0, 5, 20, or 50 μM) and sucrose (0, 5, or 10 g L^−1^). The incubation was carried out at 30 °C and 220 rpm, and the optical density at 600 nm was measured every 3 h.

**Fig. 3:**
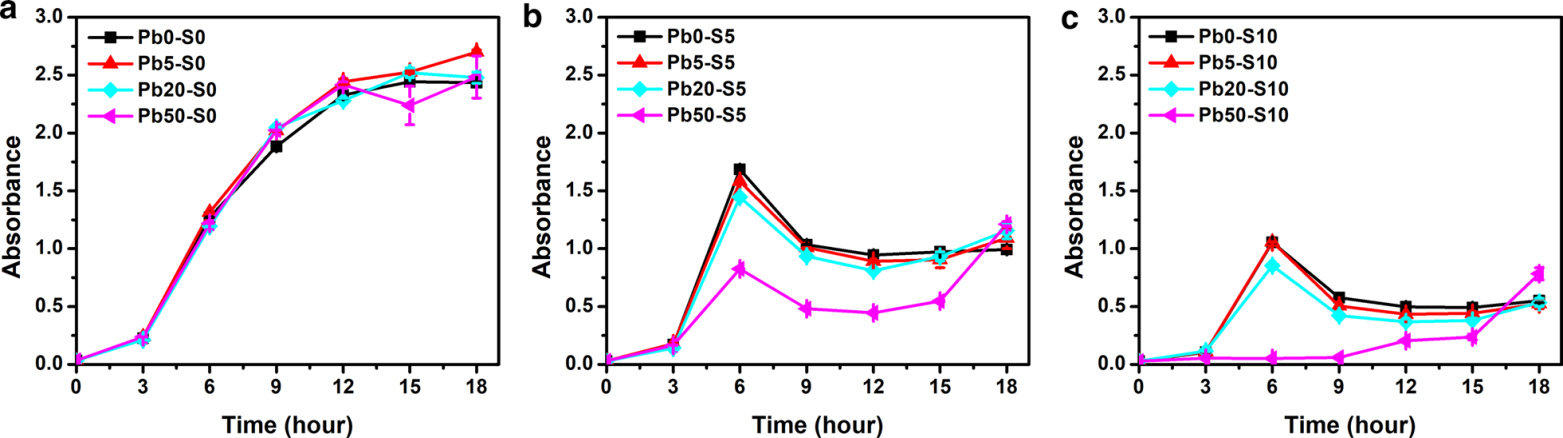
Growth curves of the chassis cells with the selection plasmid in response to lead and sucrose. The optical density at 600 nm of the *E. coli* (pZE21-PBS) strain was measured with lead at different concentrations of 0 µM (squares), 5 µM (up triangles), 20 µM (diamonds), and 50 µM (left triangles). Sucrose was used at of **a** 0 g L^−1^, **b** 10 g L^−1^, and **c** 20 g L^−1^. Experiments were performed in triplicate, and data represent the averages and standard deviations of the means

It could be seen from Fig. 3 that lead ions did not significantly inhibit cellular growth regardless of its concentration (Fig. 3a) when no sucrose was added. When the concentration of sucrose was 5 g L^−1^ (Fig. 3b), the cell growth were not affected by lead when its concentration was at 0, 5, and 20 μM, but a significant slower growth was observed with 50 μM of lead. The inhibition on cell growth by expression of *sacB* was more noticeable with a higher concentration of sucrose. When adding 10 g L^−1^ sucrose (Fig. 3c), the screened cells showed similar growth pattern with 0, 5, and 20 μM of lead. However, the overall cell density was lower compared with that at a concentration of 5 g L^−1^ sucrose. The growth of cells with 50 μM of lead and 10 g L^−1^ of sucrose was totally inhibited for the first 9 h. Therefore, sucrose was used at 10 g L^−1^ during the OFF selection stage.

#### Optimization of ion concentration

**Fig. 4:**
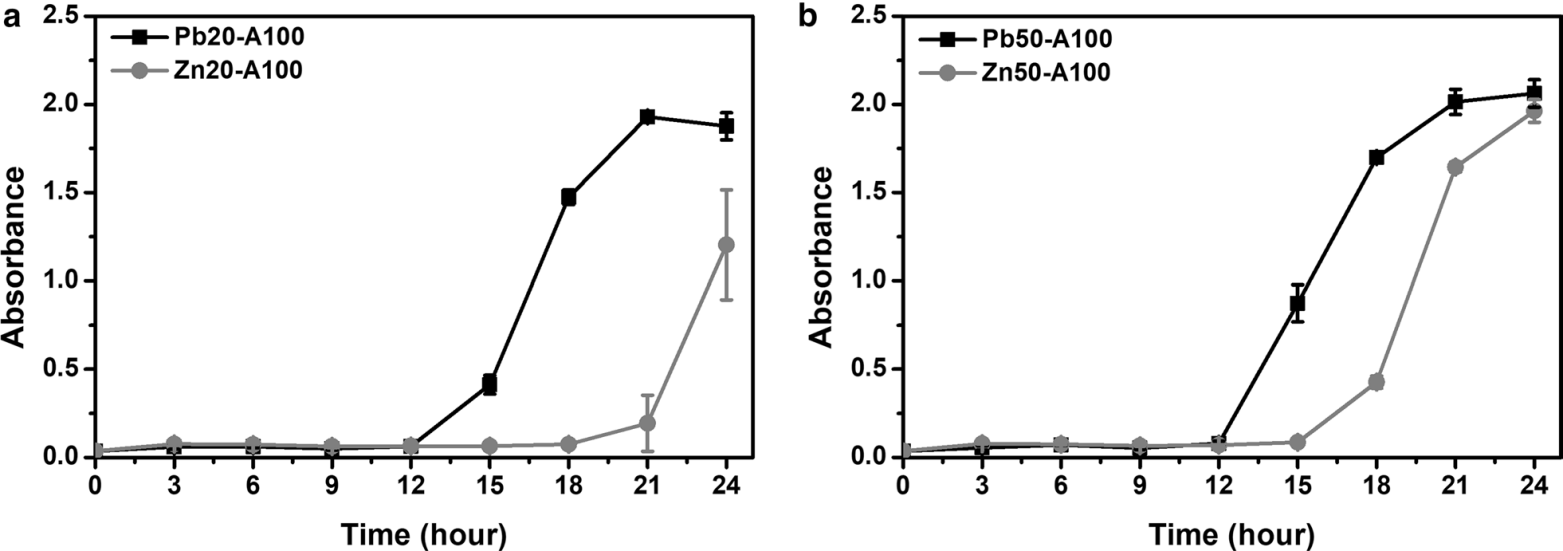
Comparison of the growth curves of the chassis cells with the selection plasmid in response to lead and zinc in the presence of ampicillin. The optical density at 600 nm of the *E. coli* (pZE21-PBS) strain was measured with metal ions at **a** 20 µM and **b** 50 µM. Ampicillin was used at 100 µg mL^−1^. Squares: lead ions; Circles: zinc ions. Experiments were performed in triplicate, and data represent the averages and standard deviations of the means

The wild-type PbrR was more specific toward lead and was expected to respond differentially to lead and zinc. The expression of the *amp* gene and the *sacB* gene was compared in response to lead and zinc at the same concentration (20 μM or 50 μM). Bacterial growth with 100 μg mL^−1^ of ampicillin at 37 °C and 220 rpm was measured to evaluate the expression of *amp* (Fig. 4). At relatively low concentration (20 μM), cells started to grow at 12 h in presence of lead and 18 h in presence of zinc, which means lead can elicit a stronger and faster expression of the *amp* gene. This difference was less when the concentration of ions increased to 50 μM. Cells grew slightly faster in presence of lead but started to grow at 15 h in presence of zinc. This was consistent with the fact that PbrR binds to zinc with a weaker affinity compared to lead.

**Fig. 5:**
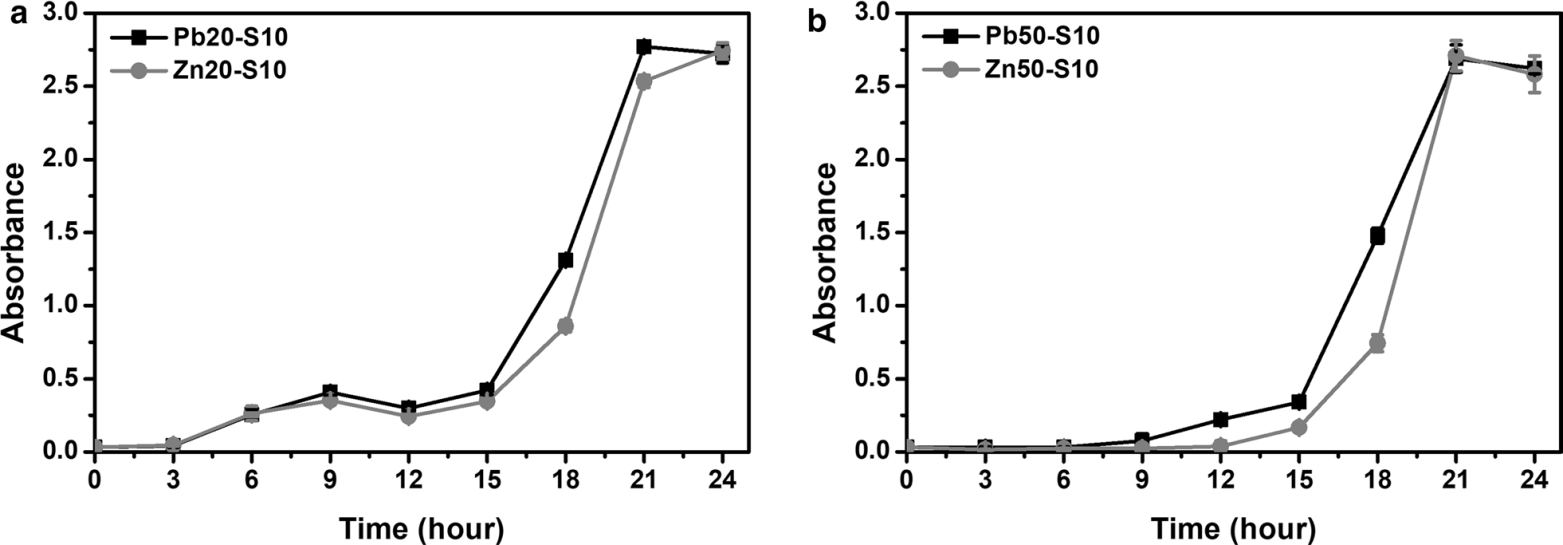
Comparison of the trowth curves of the chassis cells with the selection plasmid in response to lead and zinc in the presence of sucrose. The optical density at 600 nm of the *E. coli* (pZE21-PBS) strain was measured with metal ions at **a** 20 µM and **b** 50 µM. Sucrose was used at 10 g L^−1^. Squares: lead ions; Circles: zinc ions. Experiments were performed in triplicate, and data represent the averages and standard deviations of the means

Sucrose was used at 10 g L^−1^ at 30 °C and 220 rpm for testing the expression of *sacB* (Fig. 5). After adding sucrose, the growth patterns at the same concentration of lead and zinc were similar and a higher concentration of ions resulted in a stronger growth inhibition, though a slightly better growth was observed when lead was present.

Taken into account all these results, the selection conditions for the ON stage was set to 50 μM of lead and 100 μg mL^−1^ ampicillin, while the conditions for the OFF stage was 50 μM zinc ions and 10 g L^−1^ sucrose.

### Mutant strains

Using the selection plasmid pZE21-PBS with the *ampicillin* resistance gene and the *sacB* gene as the selection markers, directed evolution was applied to increase the specificity of PbrR toward lead. We first created a library of over 3000 *pbrR* mutants through error prone PCR in *E. coli* strain DH5α. The colonies on the transformation plates were picked and transferred to ON selection medium containing lead, as well as the OFF selection medium with zinc ions, to monitor the growth of the cells on the plate. Mutant colonies that grew on the ON selection solid medium containing lead ions and on OFF selection solid medium containing zinc ions were picked for sequencing. Finally, two mutant strains were obtained. Sequencing revealed that both strains had amino acid mutations in PbrR. The mutation of the mutant strain M1 occurred in the C-terminal metal binding domain of PbrR with a single mutation C134R from UGU to CGU, and the mutation of M2 occurred in the N-terminal dimer interface of PbrR with double mutations D64A from GAC to GCC and L68S from UUA to UCA.

### Characterization of the mutant strains

**Fig. 6:**
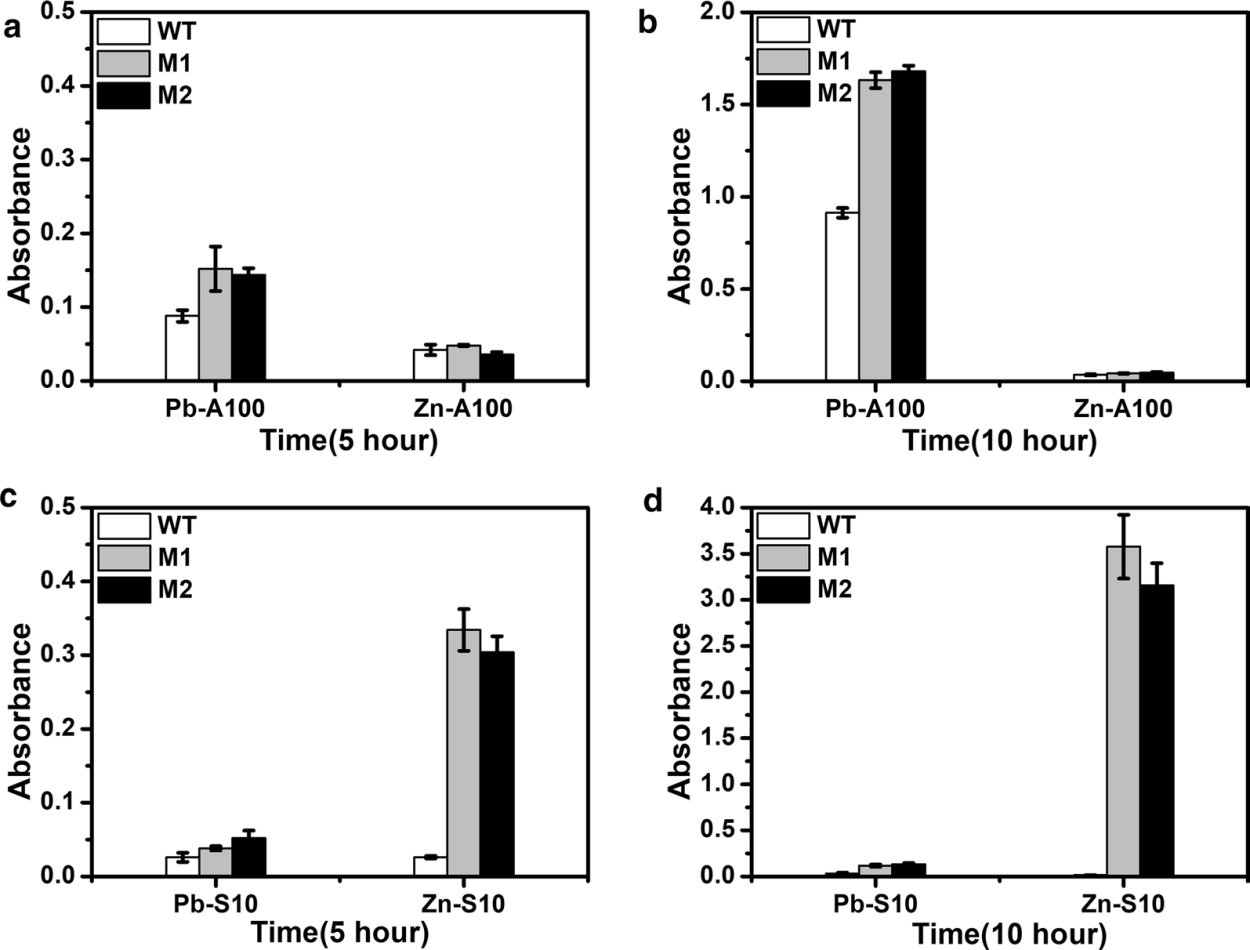
Characterization of the growth of the mutants in response to lead and zinc under the ON condition with ampicillin for **a** 5 h and **b** 10 h, under the OFF condition with sucrose for **c** 5 h and **d** 10 h. White bar: Wild type; Grey bar: Mutant M1; Black bar: Mutant M2. Culture conditions are indicated as follows: Pb—lead; A100—ampicillin; Zn—zinc; S10—sucrose. Both lead and zinc were used at 50 µM. Ampicillin was used at 100 µg mL^−1^ and sucrose was used at 10 g L^−1^. Experiments were performed in triplicate, and data represent the averages and standard deviations of the means

To evaluate the specificity of the mutants M1 and M2 toward lead, the growth of these two mutants was compared to the strain with the wild type PbrR in presence of lead or other divalent ions under selection pressures. The growth in response to lead and zinc was shown in Fig. 6. Under the ON condition with ampicillin (Pb-A100), both mutants M1 and M2 grew better than the wild type in presence of lead, showing an OD_600_ about 1.8 times of the wild type strain, which means the two PbrR mutants elicited a stronger output in response to lead compared to the wild type. Their response to zinc under the ON condition (Zn-A100) was comparable to the wild type with negligible growth for all. However, the difference in response to zinc was obvious under the OFF condition with sucrose (Zn-S10). The wild type strain cannot grow with sucrose in presence of zinc, but the two mutants showed a much better growth. The OD_600_ of the mutant M1 was about 12.8 times of that of the wild type after 5 h and 255.4 times after 10 h, and mutant M2 showed an OD_600_ about 11.7 times of the wild type after 5 h and 225.4 times after 10 h. It is clear that the two mutants are less responsive to zinc with much lower expression of *sacB*, leading to rapid growth with sucrose. The specificity of the two PbrR mutants toward lead is successfully improved compared to zinc.

**Fig. 7:**
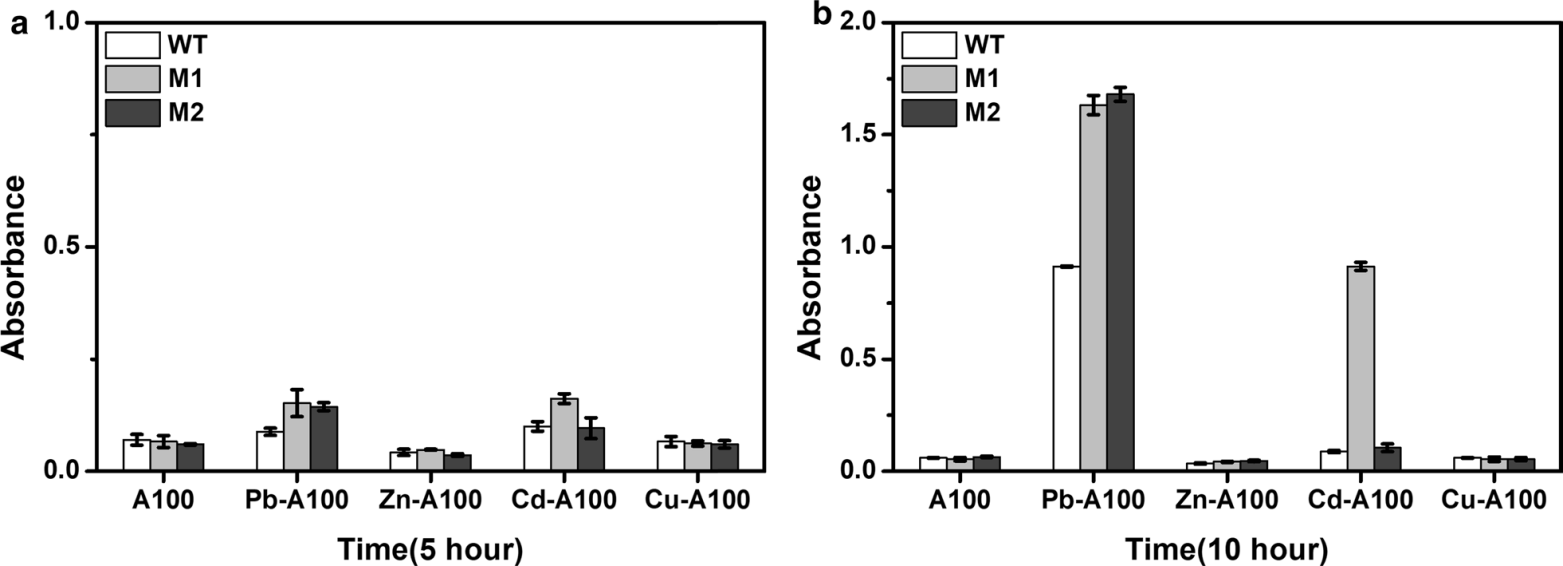
Comparison of the growth of the mutants in response to lead, zinc, cadmium, and copper in presence of ampicillin for **a** 5 h and **b** 10 h. White bar: Wild type; Grey bar: Mutant M1; Black bar: Mutant M2. Metals were used at 50 µM. Ampicillin was used at 100 µg mL^−1^. Experiments were performed in triplicate, and data represent the averages and standard deviations of the means

The response of the mutants to other commonly seen divalent heavy metal ions, copper and cadmium, were also evaluated and compared to the wild type. The growth of all strains in presence of ampicillin without any heavy metal ions was used as the control. As shown in Fig. 7, in presence of ampicillin, both mutants showed a better growth in response to lead, and similar or slightly slower growth in response to zinc and copper. However, they demonstrated different behavior in response to cadmium. Mutant M1 grew well in presence of cadmium with an OD_600_ about 10.4 times of the wild type after 10 h, but mutant M2 was comparable to the wild type. Overall, using the dual selection system, we obtained a mutant M2 that is very specific toward lead with no response from other divalent heavy metal ions tested in this study. A mutant M1 was also obtained with improved response to both lead and cadmium.

PbrR and the cadmium binding transcription factor CadR are from the same branch of the phylogenetic tree and share similar metal binding sites. Homban et al. found that cysteines C14, C79, and C134 were essential for lead ions induced transcriptional activation of P_*pbrA*_ by PbrR (Hobman et al. 2012). The mutation of the cysteine at position 134 to arginine in M1 seems to directly affect the metal recognition and binding affinity and make it more favorable to cadmium.

#### Structure simulation of the mutants

**Fig. 8:**
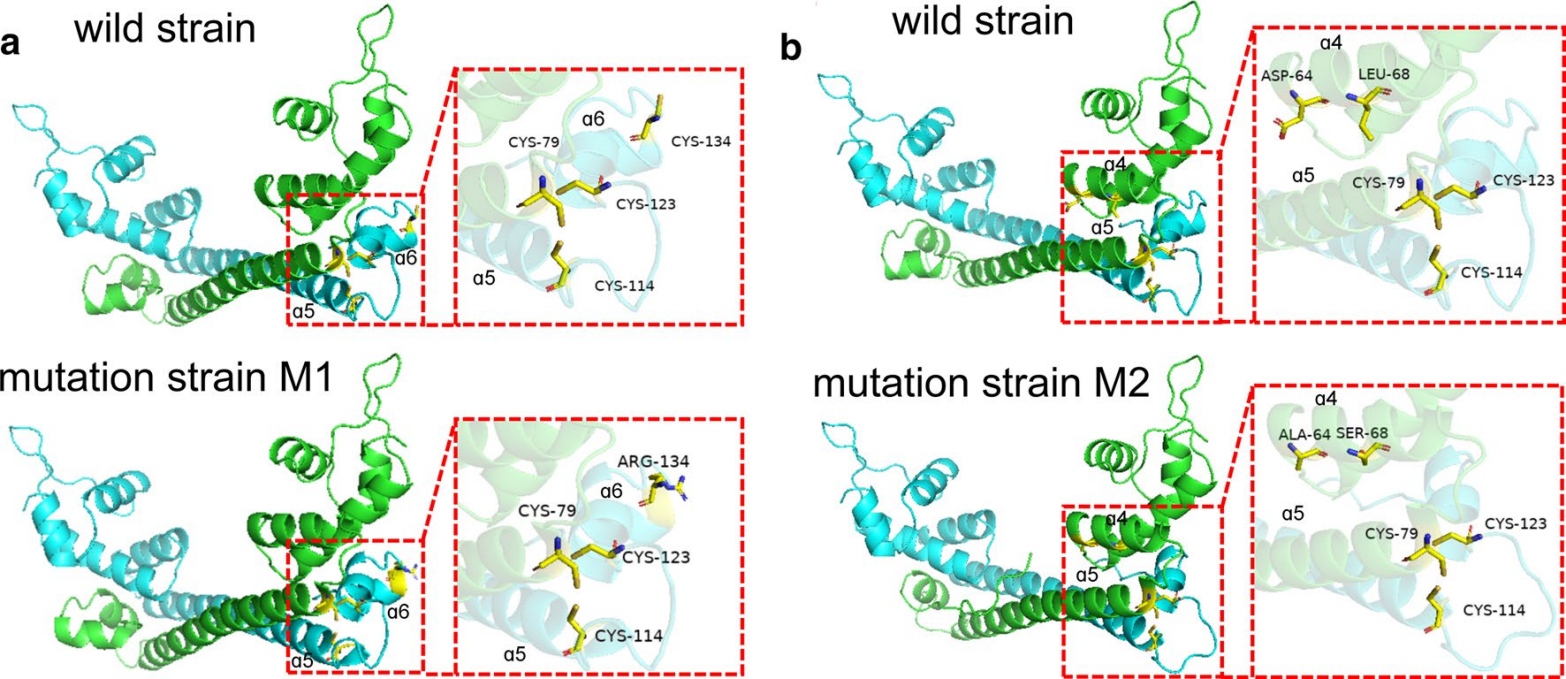
Local model comparison of protein PbrR in **a** mutant strain M1 and **b** mutant strain M2

The two mutants have different mutations with a single mutation C134R in M1 and double mutations D64A and L68S in M2. To understand the effect of the mutations on the function of the PbrR, we simulated the structure of the mutants and compared it with the wild type PbrR (Fig. 8). Sequence alignment of PbrR and other MerR type transcription factors revealed three conserved cysteine residues, C79, C114, and C123 in PbrR, coordinating metal binding and three conserved arginines, R20, R33, and R39 facilitating DNA binding and distortion of the duplex into an active conformation (Brown et al. 2003). These key residues stayed the same in both mutants so they maintained the metal ions responsive and DNA regulating functions. As shown in the simulated local model of this site shown in Fig. 8a, the metal binding cysteine residues C114 and C123 are located in the metal-binding loop region connecting helices α5 and α6, and C134 is also in the loop region. The two mutations in M2, D64A and L68S, are in the dimer interface, that is, the α-helix α4 near the loop region where C79 is located. The aspartate at position 64 of PbrR is not conserved, but the leucine at position 68 is conserved and mutated to a hydroxyloid amino acid, which may affect the hydrogen bonding and binding affinities to metal ions. Overall, the mutated amino acids are all near the metal binding domain of the dimeric protein, which cause subtle force changes and spatial changes, leading to reduced binding capacity of zinc ions.

## Discussion

Metal-binding transcription factors, such as those in the MerR family in response to divalent ions, are commonly used in whole-cell heavy metal biosensors, and their specificity determines the accuracy of the sensors. However, because of the similarity in their structure and regulatory mechanism as well as the divalent metal ions, the crosstalk between the metal-binding transcription factor and different metals severely affect the specificity of the sensors. Often the output of the sensors is attributed to different ions together and cannot be linked to the concentration of a single ion, which is one of the bottlenecks for the real application of whole-cell biosensors for heavy metal detection. PbrR is the most specific lead sensing transcription factor discovered so far and has been used in lead whole-cell biosensors. Still, the response of PbrR based biosensor to divalent ions other than lead is about 20% of that to lead. It is hard to rationally mutate it due to our poor understanding of the interaction between PbrR and different ions and the fact that these ions are very similar and bind to the same key amino acids in PbrR. Directed evolution is a powerful tool to obtain mutants with desired functions and could be used to improve the selectivity of PbrR toward lead as long as there is an effective selection method.

At present, some ingenious selection systems have been designed for the directed evolution of transcriptional regulatory proteins, and have had their compound binding specificity successfully modified. Yokobayashi et al. designed a genetic screening module to select the positive and negative expression of a gene that was coupled with survival or death of the host cell by controlling the expression of the *tetA* and *bla* resistance genes(Yokobayashi and Arnold 2005). The research team proved the practicality of this gene-screening module, but the strategy was only valid for circuits with a clear ON/OFF output. Moreover, there were more false positives in larger libraries.

In recent years, the Red positive and negative two-step screening method to achieve a gene knockout or knock-in screening strategy has attracted the attention of researchers and also promoted the understanding and development of negative screening markers. The most commonly used negative screening markers are the genes that confer sucrose, streptomycin, or fusaric acid sensitivity, while some negative screening genes require specific host cells to function. The *Bacillus subtilis sacB* gene encoding levansucrase is the most popular negative screening marker (Reyrat et al. 1998), and it converts sucrose to levans that are harmful to gram-negative bacteria. Therefore, the ampicillin resistance gene *amp* and levansucrase gene *sacB* were selected as the positive and negative marker genes, respectively, in the dual selection system of this study. The desired features of mutants, which were responsive to lead but not other ions, were coupled to fast cell growth in both positive and negative selections.

In this study, we constructed a novel dual selection plasmid pZE21-PBS, where *pbrR* and the genes for selection were located on the opposite sides of the bidirectional promoter from the *pbr* operon (Fig. 1). The expression of two selection genes was controlled by the transcription factor PbrR and its promoter P_*pbr*_. ON selection with the lead ions and OFF selection with the zinc ions were successively carried out to select mutants that are only responsive to lead. Two mutants M1 and M2 with fast growth at both stages were obtained. Both mutants showed a stronger response to lead. The OD_600_ was 1.8 times and 2 times higher than that of wild type. The OD_600_ also increased under the OFF condition in the presence of zinc ions, indicating that their response to zinc was weakened. The results indicated that the dual selection system with the ampicillin resistance gene *amp* as the positive marker and the levansucrasegene *sacB* as the negative marker was effective for the selection of mutants of transcription factors with enhanced specificity toward an analyte. This dual selection system can be used for the directed evolution of many other transcription factors to improve the specificity toward an inducer, not just heavy metal ions. The PbrR regulatory element is a good example of the one-component regulatory systems. The success with PbrR indicates that this system can be adapted to other one-component systems like regulators in response to antibiotics or sugars. In addition, it can be used to improve the specificity of regulators in two-component regulatory systems, which are very common in bacteria, such as the complex chemotaxis pathway in response to nutrients like amino acids. They will provide not only regulatory elements with high specificity for biosensing but also valuable tools in other areas of synthetic biology.

## Supplementary information

**Additional file 1.** Additional tables.

## Data Availability

Corresponding author could provide the all experimental data on valid request.
