# Supplementary material for: Directed evolution of a transcription factor PbrR to improve lead selectivity and reduce zinc interference through dual selection
Source: AMB Express. 2020 Apr 10;10:67. doi: 10.1186/s13568-020-01004-8 (PMC7148400; doi:10.1186/s13568-020-01004-8)
Supplement: Supplementary file 1 — Additional file 1. Additional tables. [file 13568_2020_1004_MOESM1_ESM.docx]

**Additional Material**

**Manuscript Title**

Directed evolution of a transcription factor PbrR to improve lead selectivity and reduce zinc interference through dual selection

**Journal name**

AMB Express

**The names of the authors**

Xiaoqiang Jia^﹩1,2,3*^, Yubing Ma^﹩1^, Rongrong Bu^﹩1^, Tingting Zhao^1^, and Kang Wu^4*^

**The affiliations and addresses of the authors**

1 Department of Biochemical Engineering, School of Chemical Engineering and Technology, Tianjin University, Tianjin 300072, PR China

2 Frontier Science Center for Synthetic Biology and Key Laboratory of Systems Bioengineering (MOE), School of Chemical Engineering and Technology, Tianjin University, Tianjin, 300350, PR China.

3 Collaborative Innovation Center of Chemical Science and Engineering (Tianjin), Tianjin 300072, PR China

4 Department of Chemical Engineering, University of New Hampshire, Durham NH 03824, USA

**The e-mail address and telephone of the corresponding author**

*Corresponding author: Xiaoqiang Jia (E-mail address: xqjia@tju.edu.cn; telephone: 18920698855), Kang Wu (E-mail address: [Kang.Wu@unh.edu](mailto:Kang.Wu@unh.edu))

^﹩^Xiaoqiang Jia, Yubing Ma and Rongrong Bu should be considered joint first author

**Table S1** *Escherichia coli* strain and plasmids used in this study

| **Strain/plasmids** | **Description** | **Source** |
| --- | --- | --- |
| Strain |  |  |
| DH5α | *F- φ80lacZΔM15 Δ(lacZYA-argF)U169 deoR recA1 endA1 hsdR17(rk-, mk+) phoA supE44 λ- thi-1 gyrA96 relA1* | Invitrogen |
| Plasmids |  |  |
| pZE21 | P_LtetO-1_ MCS1 ColE1 *kan* | (Lutz and Bujard 1997) |
| pUC57-G7-kan | kan ΔpbrA P_pbrRT_ pbrR ter ΔpbrR P_pbrABCD_ | (Jia et al. 2018) |
| pZE12 | P_LlacO-1_ MCS1 ColE1 *amp* | (Lutz and Bujard 1997) |
| pK18mobsacB | Suicide vector for gene deletion in *P. putida*, *kan* | (Schafer et al. 1994) |
| pZE21-PBS | *kan pbrR* P*_pbr_* *amp sacB* ColE1 *kan* | This study |

**Table S2.** List of primers used in this study

| **Primer name** | **Sequence (from 5’ to 3’)** |
| --- | --- |
| H1F | GGCATCAAATAAAACGAAAGGCTC |
| H1R | GACGTCGGAATTGCCAGCTG |
| H2F | CAGCTGGCAATTCCGACGTCTCTAGGGCGGCGGATTTGTC |
| H2R | CGGAAATGTTGAATACTCATGGCAACCCCTTGTGTGTATTC |
| H3F | ATGAGTATTCAACATTTCCGTGTCG |
| H3R | TTCATTAATTTCTCCTCTTTCGGTTACCAATGCTTAATCAGTG |
| H4F | CCGAAAGAGGAGAAATTAATGAACATCAAAAAGTTTGC |
| H4R | AGCCTTTCGTTTTATTTGATGCCTTATTTGTTAACTGTTAATTG |
| EP2F | CTTTCGTTTTATTTGATGCCCTAGTCGCTTGGATGGGCG |
| EP2R | GACATCTCCCATCCGACGCCATGAATATCCAGATCGGCGAG |
| EPF | GGCGTCGGATGGGAGATGTC |
| EPR | GGCATCAAATAAAACGAAAGGC |
| SP12F | GCGGCAAGAAAGCCATCCAGTTTAC |
| SP23R | CAAAAAAGGGAATAAGGGCGACACG |
